# Caveolin-1 Provides Palliation for Adverse Hepatic Reactions in Hypercholesterolemic Rabbits

**DOI:** 10.1371/journal.pone.0071862

**Published:** 2014-01-24

**Authors:** Ya-Hui Chen, Wei-Wen Lin, Chin-San Liu, Li-Sung Hsu, Yueh-Min Lin, Shih-Li Su

**Affiliations:** 1 Vascular and Genomic Center, Changhua Christian Hospital, Changhua, Taiwan; 2 Institute of Biochemistry and Biotechnology, Chung Shan Medical University, Taichung, Taiwan; 3 Division of Cardiovascular Center, Department of Internal Medicine, Taichung Veterans General Hospital, Taichung, Taiwan; 4 Graduate Institute of Integrative Medicine, China Medical University, Taichung, Taiwan; 5 Department of Pathology, Changhua Christian Hospital, Changhua, Taiwan; 6 Department of Medical Technology, Jen-Teh Junior College of Medicine, Nursing and Management, Miaoli, Taiwan; 7 Division of Endocrinology and Metabolism, Department of Internal Medicine, Changhua Christian Hospital, Changhua, Taiwan; 8 Institute of Medicine, Chung Shan Medical University, Taichung, Taiwan; Institute of Molecular and Cell Biology, Biopolis, United States of America

## Abstract

Caveolins are an essential component of cholesterol-rich invaginations of the plasma membrane known as caveolae. These flask-shaped, invaginated structures participate in a number of important cellular processes, including vesicular transport, cholesterol homeostasis, and signal transduction. We investigated the effects of CAV-1 on mitochondrial biogenesis and antioxidant enzymes in hypercholesterolemia-affected target organs. A total of eighteen male New Zealand white rabbits were divided into three groups: a normal-diet group, an untreated hypercholesterolemia-induced group, and a hypercholesterolemia-induced group that received intravenous administration of antennapedia-CAV-1 (AP-CAV-1) peptide every 2 days for 2 weeks. Serum biochemistry, CAV-1 distribution, neutral lipid distribution, mitochondrial morphology, biogenesis-mediated protein content, oxidative stress balance, antioxidant enzyme levels, and apoptotic cell death of liver tissue were analysed. Hepatic and circulating cholesterol and low-density lipoprotein cholesterol (LDL-C) levels differed significantly between the three groups (*P*<0.05). Immunohistochemical staining intensity of CAV-1 was greater in AP-CAV-1-treated rabbits than in untreated rabbits, especially in the vicinity of the liver vasculature. The high levels of neutral lipids, malondialdehyde, peroxisome proliferator-activated receptor-γ coactive 1α (PGC-1α), and nuclear respiratory factor-1 (NRF-1) seen in untreated hypercholesteremic animals were attenuated by administration of AP-CAV-1 (*P*<0.05). In addition, mitochondria in animals that received treatment exhibited darker electron-dense matrix and integrated cristae. Furthermore, the levels of ROS modulator 1 (Romo1) and superoxide dismutase (SOD)-2, as well as catalase activity were significantly lower in CAV-1-treated hypercholesterolemic rabbits (*P*<0.05). AP-CAV-1 treatment also restored mitochondrial respiratory chain subunit protein content (OXPHOS complexes I–V), thereby preserving mitochondrial function (*P*<0.05). Furthermore, AP-CAV-1 treatment significantly suppressed apoptotic cell death, as evidenced by a reduction in the number of TUNEL-positive cells. Our results indirectly indicate that CAV-1 mediates the negative effects of PGC-1α on hepatic mitochondrial respiratory chain function, promotes the antioxidant enzyme defence system, and maintains mitochondrial biogenesis.

## Introduction

Caveolae are omega-shaped invaginations of the plasma membrane with a diameter of approximately 50–100 nm. These flask-shaped, invaginated structures participate in a number of important cellular processes including vesicular transport, cholesterol homeostasis, signal transduction and tumour suppression [1], [2]. They also play a prominent role in various human pathobiological conditions, particularly in the cardiovascular system [3]–[5]. The key structural and functional protein in caveolae in most cell types is caveolin (CAV). Deficiency in CAV-1, an important protein marker of caveolae, has been shown to result in cholesterol ester uptake associated with increased acyl-CoA:cholesterol acyl-transferase activity and scavenger receptor class B type I (SR-BI) overexpression [6]–[8]. Frank *et al*., found that caveolin-1-deficient (CAV-1 ^−/−^) mice exhibited enhanced plasma triglyceride (TG) levels, increased high-density lipoprotein (HDL) levels, and reduced hepatic very low-density lipoprotein (VLDL) secretion. They also found that caveolin-1 deficiency prevented transcytosis of LDL across endothelial cells, suggesting that CAV-1 may be implicated in the regulation of plasma LDL levels and, hence, may play an important role in the development of atherosclerosis [2]. In CAV-1-overexpressing animal models, plasma HDL-cholesterol (HDL-C) levels and hepatic free cholesterol levels were elevated, but TG and total cholesterol levels remained unchanged. In addition, CAV-1 has been shown to stimulate HDL-mediated cellular cholesterol efflux, which further prevents ox-LDL uptake into human endothelial cells [9]–[12]. The effect of CAV-1 expression on cholesterol efflux remains controversial. A number of studies have shown that CAV-1 deficiency results in accumulation of free cholesterol in mitochondrial membranes, which causes mitochondrial dysfunction and eventually cell death [1], [13]–[16]. Studies have also shown that CAV-1 negatively regulates endothelial nitric oxide synthase (eNOS), the enzyme responsible for synthesizing nitric oxide [4], [17]–[19].

More than 90% of cellular ATP generation occurs in the mitochondria via oxidative phosphorylation (OXPHOS). In addition, mitochondria are principally responsible for the generation of reactive oxygen species (ROS), calcium homeostasis, stimulation of apoptosis, and ageing [20], [21]. The peroxisome proliferator-activated receptor-γ coactive 1α (PGC-1α) is a major regulator of mitochondrial biogenesis and function-related gene expression [i.e. nuclear respiratory factor-1 (NRF-1) and mitochondrial transcription factor A]. Valle *et al.* demonstrated that PGC-1α overexpression resulted in a notable reduction in ROS accumulation, upregulation of mitochondrial number and activity and reduction in apoptotic cell death, even under oxidative stress conditions, leading to protection against vascular endothelial cell dysfunction, particularly in various cardiovascular diseases [22], [23]. Studies have shown that changes in mitochondrial biogenesis are associated with diabetic retinopathy, diabetic neuropathy, chronic exercise, inflammatory processes, chronic cholestasis, and cholestatic liver diseases [24]–[31]. However, there is no clear therapeutic strategy for improving mitochondrial dysfunction and enhancing clinical outcome [32].

Antennapedia-CAV-1 peptide, a homeodomain of antennapedia, is a Drosophila transcription factor that can be internalized by cells via a receptor-independent pathway. It possesses translocating properties and is capable of carrying peptides comprising 20–30 amino acid residues across the plasma membrane [33]–[34]. Coupling of the CAV-1 C–terminal scaffolding domain (amino acids 82–101) to the AP peptide was shown to facilitate its translocation across the cell membrane in vivo. After in vivo delivery, AP-CAV-1 attenuated eNOS-dependent NO production and reduced inflammation in mice. AP-CAV-1 also inhibited smooth muscle proliferation in mice with pulmonary artery hypertension [35]–[36]. Our previous study revealed that CAV-1 may be important in the prevention and treatment of atherosclerosis [37]. In addition, CAV-1 is associated with the HDL-mediated cholesterol efflux pathway in aortic endothelial cells [38]. Whether this effect of CAV-1 is related to the disturbance of oxidative and antioxidative homeostasis, mitochondrial biogenesis, and mitochondrial activity remains unknown. Therefore, in the present study, we investigated the effects of CAV-1 on cholesterol efflux into the circulation, neutral lipid distribution, mitochondrial function, mitochondrial morphology, mitochondrial biogenesis, apoptotic cell death, and oxidative stress balance in a rabbit model of hypercholesterolemia.

## Materials and Methods

### Animals and DNA Extraction

A total of eighteen male New Zealand white rabbits (weighing approximately 4 kg) were divided into three groups. Rabbits in the first group (normal-diet group, n = 6) were fed a standard rabbit chow diet (Fu-Shou Co.; Taichung, Taiwan). Animals in the second group (untreated hypercholesterolemia-induced group, n = 6) were fed a 2% cholesterol diet for 8 weeks. Rabbits in the third group (treated hypercholesterolaemia-induced group, n = 6) were fed a 2% cholesterol diet for 8 weeks and then, beginning on the sixth week, received intravenous administration of antennapedia-CAV-1 (AP-CAV-1) peptide [RQPKIWEFPNRRKPWKK-DGIWKASFTTFVTKYWFYR-(OH); GU-Yuan Biotech Services Corp., TW; 1 mg/kg] every 2 days for 2 weeks. The rabbits in each group were sacrificed after 8 weeks. Blood and liver tissue were collected for analysis. Hepatic DNA was isolated using GenoMarker reagent according to the manufacturer's recommendations. The animal facilities and protocols were reviewed and approved by the Institutional Animal Care and Use Committee (IACUC) of the Taichung Veterans General Hospital, Taichung, Taiwan (Approval No: La-95278).

### Analysis of Serum Biochemistry

Blood (3.0 ml) was collected from rabbit ears into vacutainer® vials and centrifuged at 3000 rpm for 10 min. Total serum glucose, cholesterol, TG, AST, ALT, and free fatty acid levels were measured by enzymatic colorimetric methods using an automatic analyser (ADVIA 1800; Siemens, Tarrytown, NY, USA). HDL-C and LDL-C were measured by elimination/catalase methods using an automatic analyser (ADVIA 1800; Siemens). Insulin was detected by a chemiluminescence method using an autoanalyser (ADVA Centaur; Siemens).

### Primer Design and Real-Time PCR

Sequences of rabbit mtDNA (GeneBank accession number, NC_001913) and β-actin (BA) (GeneBank accession number, NW_003159464) were used to design species-specific primers. The rabbit mtDNA D-loop (DL)-specific primers used in this study are as follows: forward, 5′-gg ttc tta cct cag ggc cat ga-3′and reverse, 5′-gat tag tca tta gtc cat cga gat-3′. The rabbit BA-specific primers used in this study are as follows: forward, 5′-atc gtg cgc gac atc aag gag aag c-3′ and reverse, 5′-g cag ctc gta gct ctt ctc cag-3′. PCR amplification was performed in 50 µl reaction volumes containing 5× Platinum® SYBR® Green qPCR SuperMix UDG, 10 µM each of DL or BA forward and reverse primers, 1× ROX reference dye, and approximately 50 ng of sample DNA. The cycling conditions included an initial phase of 2 min at 50°C, followed by 2 min at 95°C and 40 cycles of 15 s at 95°C and 30 s at 60°C. Each sample was assayed in duplicate, and fluorescence spectra were continuously monitored using the 7700 Sequence Detection System (Applied Biosystems). Data analysis was based on measurement of the cycle threshold (C_T_). The difference in C_T_ values was used as the measure of relative abundance, i.e. C_T_ (DL)−C_T_ (BA) was used to measure the abundance of the mitochondrial genome.

### Electron Microscopy

Liver slices (1-mm^3^ thick) were prepared manually and were immediately placed in cold fixative consisting of a mixture of 4% formaldehyde and 1% glutaraldehyde in 0.2 M cacodylate buffer (pH 7.4) for 6 h, post-fixed with osmium tetraoxide, and embedded in Spurr's resin. Ultrathin sections were double stained with uranyl acetate and lead citrate and examined using a JEM-1230 electron microscope (JEOL Ltd., Japan).

### Immunohistochemistry, Imaging and Quantification

Immunohistochemistry was performed as described previously [37]. The following primary antibodies were used: monoclonal anti-CAV-1 (1∶250; Epitomics, Burlingame, CA, USA), monoclonal anti-PGC-1α (1∶250; Abcam, Cambridge, MA, USA), monoclonal anti-reactive oxygen species modulator 1 (ROMO1) (1∶150, OriGene, Rockville, MD, USA), which has been reported to generate ROS in mitochondria [39], polyclonal anti-NRF-1 (3 µg/ml; GeneTex, Irvine, CA, USA), , polyclonal anti-SOD2 (1∶1000, Novus, Littleton, CO, USA), and polyclonal anti-catalase (1∶1000; Abcam). Nuclei were counterstained with haematoxylin and photographed using an Olympus BX61 microscope (Tokyo, Japan). For animal liver tissues, positive cells were quantified in 5 fields per animal at ×400, N = 6 rabbits for each group per time point (Image-Pro Plus 4.5).

### Immunoblot Analysis

Tissue lysate was harvested in lysis buffer (25 mM bicine, 150 mM sodium chloride, pH 7.6, Pierce), homogenized, and centrifuged for 20 min at 12,000 rpm at 4°C. The protein concentration was detected using a BCA protein assay kit (Pierce BCA assay, Thermo Scientific, Rockford, IL). Protein (30 µg) was separated by 10% or 12% SDS-PAGE and then transferred to PVDF membranes (Pall Corporation). Blots were then probed with monoclonal anti-CAV-1 (1∶1000; Epitomics, Burlingame, CA, USA), monoclonal anti-PGC-1α (1 µg/ml; Abcam, Cambridge, MA, USA), polyclonal anti-NRF-1 (1∶1000; GeneTex, Irvine, CA, USA), polyclonal anti-SOD2 (1∶1000, Novus, Littleton, CO, USA), polyclonal anti-catalase (1∶1000; Abcam), MitoProfile Total OXPHOS rodent antibody cocktail (1∶800; MitoSciences, Eugene, OR), and mouse anti-β-actin (1∶10,000; Millipore). Signals were obtained using an enhanced chemiluminescence kit (Millipore, Billerica, Massachusetts, USA) and densitometry was performed using Fusion-Capt software (Vilber Lourmat, Fusion FX7, France).

### Apoptosis Determination

Liver sections (4-µm thick) were deparaffinized with xylene, rehydrated in ethanol, and washed with PBS buffer. The sections were incubated with proteinase K (25 µg/ml) for 30 min at room temperature to expose DNA for end labelling. A positive control liver section was exposed to DNase I solution for 30 min at room temperature. After incubation, cell death was detected in liver sections by a terminal deoxynucleotidyl transferase dUTP nick end-labelling (TUNEL) assay kit (Invitrogen, Eugene, OR, USA) according to the manufacturer's instructions. The sections were examined by fluorescence microscopy using an Olympus BX61 microscope and TUNEL-positive cells were quantified in 7 fields per animal at ×400, N = 6 rabbits for each group per time point (Image-Pro Plus 4.5).

### Neutral Lipid Staining

Fresh liver tissue was frozen in optimal cutting temperature compound (Sakura Finetek, Torrance, CA, USA) and cryostat sections (7 µm-thick) were fixed in ice-cold 10% formalin for 10 min. Fresh oil-red O solution was prepared by dilution of the oil-red O stock solution (Sigma, 3.5 g/l in 99.5% isopropyl alcohol) in distilled water to a final concentration of 0.2%. This solution was mixed thoroughly and filtered through a 0.2-µm filter before use. Next, slides were placed in absolute isopropyl alcohol for 2 min. Subsequently, the sections were stained in pre-warmed oil-red O solution for 20 min in an oven at 60°C, rinsed in distilled water and counterstained with haematoxylin, followed by washing with running tap water. The slides were mounted with Clear Mount™ Mounting solution (Invitrogen, Eugene) and examined using an Olympus BX61 microscope.

### Malondialdehyde (MDA) Measurement

MDA formation, a measure of lipid peroxidation, was assayed by the reaction with thiobarbituric acid (TBA). MDA concentration was detected in liver supernatant by a NWLSS™ Malondialdehyde assay kit (Northwest, Vancouver, WA, USA).

In brief, we prepared a 10% w/v homogenate in cold assay buffer via centrifugation and stored the resultant supernatant on ice. Then, BHT reagent (5 µl), acid reagent (125 µl), and TBA reagent (125 µl) were added to 125 µl of tissue supernatant and the mixture was incubated at 60°C for 1 h. After cooling the vials at 4°C for 10 min the mixture was centrifuged at 10,000× *g* for 2–3 min. A 200-µl reaction mixture was then transferred to a 96-well microplate and absorbance was measured at 532 nm. The results are expressed as nanomoles of malondialdehyde per milligram of protein.

### Statistical Analysis

Statistical analyses were performed using one-way ANOVA and SigmaPlot *t*-tests. Data are presented as mean ± SD from 3 independent experiments. A *P* value<0.05 was considered to represent statistical significance.

## Results

### Serum Biochemistry

The serum glucose, cholesterol, and LDL-C levels differed significantly among each group (*P<*0.05; Table 1). Blood glucose and cholesterol levels were significantly higher in untreated hypercholesterolemic rabbits (untreated control group) and in caveolin-1-treated rabbits (CAV-1 group) than in rabbits that received a diet of normal rat chow (normal-diet group). LDL-C levels were also significantly higher in the hypercholesterolemic rabbits than in rabbits that were fed a normal diet (*P*<0.05). In addition, glucose, cholesterol, TG, AST, ALT, LDL-C, insulin, and free fatty acid levels were significantly lower in the CAV-1 group than in the untreated group.

**Table 1:** Characteristics of the experimental animals.

Parameters^a^^, ^ b	Normal	Control	Caveolin-1	*P-value*
Glucose, mg/dL	124.3±1.3	110.3±2.9*	104.0±4.0*	0.007
Cholesterol, mg/dL	41.67±4.67	665.0±26.9*	659.3±167.8*	0.006
TG, mg/dL	40.67±3.18	72.33±13.53	52.00±4.72	0.095
AST, U/L	37.67±0.88	118.67±80.25	51.33±7.69	0.467
ALT, U/L	46.00±6.03	64.67±31.23	43.33±8.67	0.704
HDL-C, mg/dL	23.33±4.33	27.00±0.58	26.33±3.76	0.722
LDL-C, mg/dL	9.67±0.33	248.7±9.8*	211.7±85.1	0.029
INSbay, mU/L	28.37±11.46	20.10±5.32	13.77±5.26	0.470
FFA, mmol/L	0.19±0.01	0.40±0.10	0.33±0.02	0.102

a , Data are represented as mean ± SD obtained from three independent experiments, n = 6.

b , Normal group: normal diet without any treatment; Control group: fed 2% cholesterol diet; Caveolin-1 group:

fed 2% cholesterol diet and treated with caveolin-1 peptide. TG, triglyceride; AST, aspartate aminotransferase; ALT, alanine transaminase; HDL-C, high-density lipoprotein cholesterol; LDL-C, low-density lipoprotein cholesterol; INSbay, insulin; FFA, free-fatty acid.

Data were analyzed by one-way ANOVA.

* , p<0.05; the results was compared to the normal group.

### Distribution of CAV-1 and Neutral Lipids in Hepatic Cells of Hypercholesterolemic rabbits

**Figure 1:**
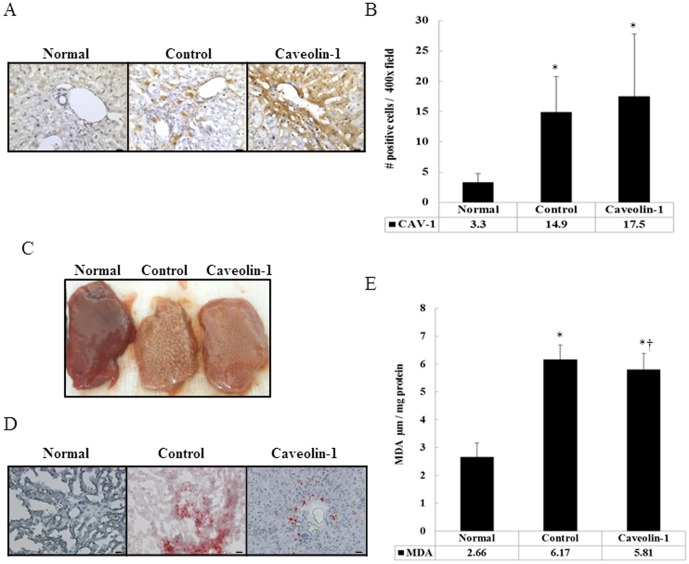
CAV-1 distribution and neutral lipid accumulation in liver tissue after 8 weeks on the normal diet and high-cholesterol diet with or without CAV-1 treatment. (A) Note that CAV-1 is mainly expressed in the vicinity of the liver vasculature. Staining intensity was greater in CAV-1-treated rabbits (CAV-1 group) than in untreated hypercholesterolemic rabbits (control group). (B) Quantification of CAV-1 IHC staining in rabbit liver tissue per ×400 field/5 fields per animal, n = 6 rabbits for each group. Neutral lipid accumulation in the liver cells, indicated by exterior images (C) and oil-red O staining (D), was significantly higher in the control group than in the CAV-1 group. (E) High-cholesterol diet induced a significant increase in malondialdehyde levels in liver tissue. The data represent those from at least 3 independent experiments (magnification 400×, n = 6 for each group, Bar = 1 µm). * and † are significantly different from the normal and control groups, respectively, *P*<.05.

Analysis of liver sections revealed that the staining intensity of CAV-1 was higher in hypercholesterolemic rabbits than in the normal-diet group (14.9±5.9 and 3.3±1.48 positive cells/400× field, *P*<0.05). Our previous study indicated that CAV-1 expression in the thoracic aorta of rabbits fed a high-cholesterol diet reaches its highest level at 5 weeks and then gradually decreases after 8 weeks [37]. Thus, hypercholesterolemic rabbits were administered intravenous injections of AP-CAV-1, 1 mg/kg, every 2 days for 2 weeks. Comparison of CAV-1 distribution in the CAV-1 group with that in the control group (17.5±10.3 and 14.9±5.9 positive cells/400× field) is shown in Figure 1A and 1B. Hepatic cells in the control group showed a markedly higher degree of oil-red O staining and significantly higher malondialdehyde levels (6.17±0.51 µM/mg protein, *P*<0.05), a reflection of neutral lipid accumulation, than hepatic cells in the AP-CAV-1-treated group and the normal-diet group (Figure 1C–1E).

### Mitochondrial Function, Biogenesis, and Morphology

**Figure 2:**
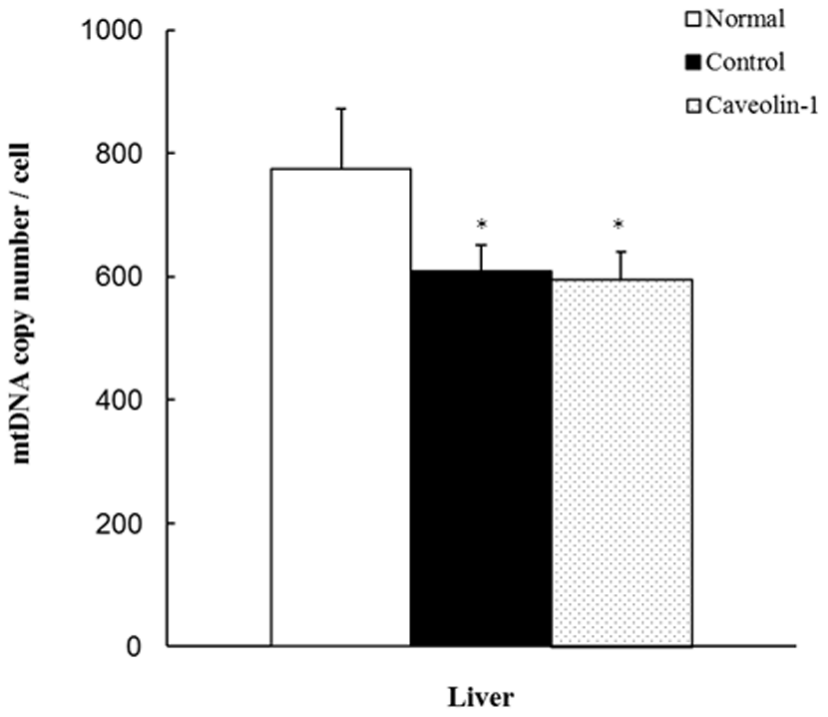
Changes in the mtDNA copy numbers in the control and CAV-1 groups. A significant decrease in mtDNA copy number/cell was observed in the CAV-1 group compared with the normal group by SigmaPlot *t*-tests (* *P<*0.05). Values are mean ± SD from at least 3 independent experiments, n = 6 for each group.

**Figure 3:**
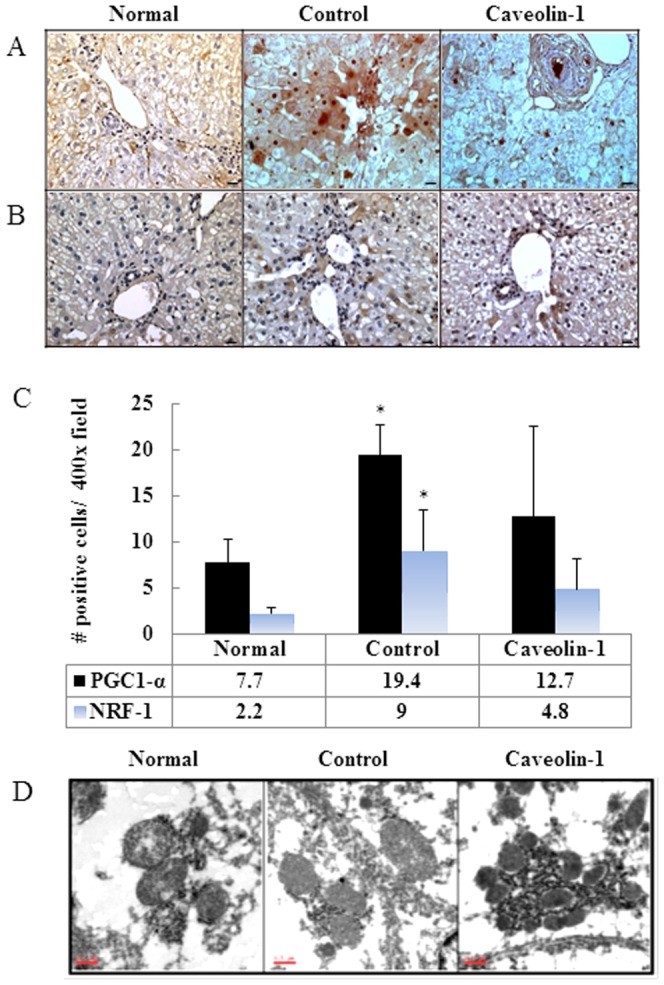
CAV-1 reduces mitochondrial biogenesis and rescues morphological abnormalities in hypercholesterolaemic rabbits. Expression levels of PGC-1α (A) and NRF-1 (B) proteins were higher in the control group but not in the CAV-1 group (magnification 400×, Bar = 1 µm). (C) Quantification of mitochondrial biogenesis marker PGC-1α and NRF-1 IHC stain per ×400 field/5 fields per animal, n = 6 rabbits for each group. * *P*<.05 compared with normal group. (D) Electron microscopy reveals a more condensed cytoplasm, with a dark electron-dense matrix and integrated cristae after CAV-1 treatment (magnification 3000×, n = 6 for each group, Bar = 0.5 µm). The data represent those from at least 3 independent experiments.

The mtDNA copy numbers, PGC-1α and NRF-1 expression levels, as well as mitochondrial morphology in rabbit liver were examined. The real-time PCR assay revealed that the mtDNA copy number/cell in the control and CAV-1 groups was significantly lower than that in the normal-diet group (*P*<0.05) (Figure 2). Immunohistochemistry revealed markedly higher levels of PGC-1α and NRF-1 in the livers of the hypercholesterolemic rabbits than in livers of the normal-diet group. However, at the end of the experiment, expression levels of PGC-1 α and NRF-1 were 0.4- and 0.5-fold lower, respectively, in the AP-CAV-1-treated group than in the untreated group (Figure 3A–3C). In addition, electron microscopy revealed a more condensed cytoplasm, with a dark electron-dense matrix and integrated cristae after CAV-1 treatment (Figure 3D).

### Romo1-derived ROS and Antioxidant Enzymes Expression

**Figure 4:**
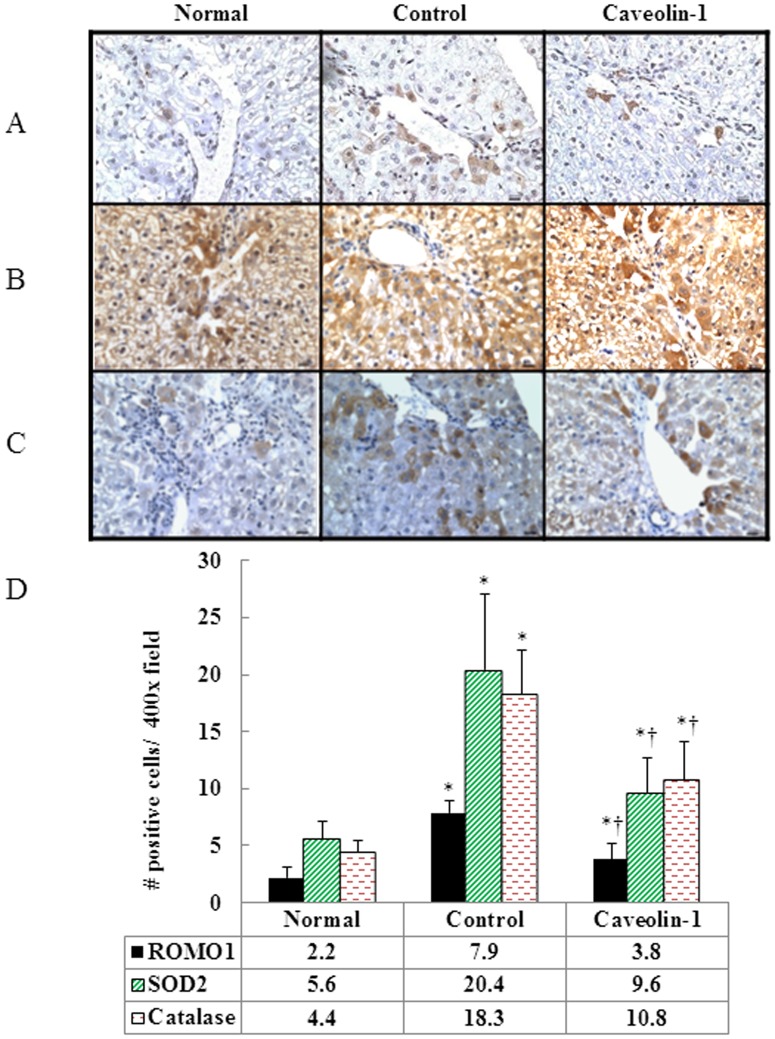
CAV-1 expression prevents Romo1-derived-ROS generation and increases the activity of antioxidant proteins. (A) Levels of the ROS modulator 1 (Romo1), mitochondrial antioxidant enzymes SOD2 (B) and catalase (C) were detected by immunohistochemistry. AP-CAV-1 treatment resulted in reduced expression of Romo1 protein and activity of SOD2 and catalase. (D) Quantification of Romo1, SOD2 and catalase staining. * and † are significantly different from the normal and control groups, respectively, *P*<.05. The data represent those from at least 3 independent experiments (magnification 400×, n = 6 for each group, Bar = 1 µm).

We then examined whether AP-CAV-1 treatment has an attenuation effect on the increased levels of mitochondrial-derived ROS seen in hypercholesterolemic rabbits. We found that the level of Romo-1, a novel source of ROS in mitochondria, was 1.1-fold lower in the CAV-1 group than in untreated hypercholesterolemic animals. In addition, AP-CAV-1 treatment resulted in a 1.1-fold reduction in SOD2 and a 0.7-fold reduction in catalase activity (*P*<0.05) (Figure 4A–4D).

### Mitochondrial Respiratory Chain Subunits

**Figure 5:**
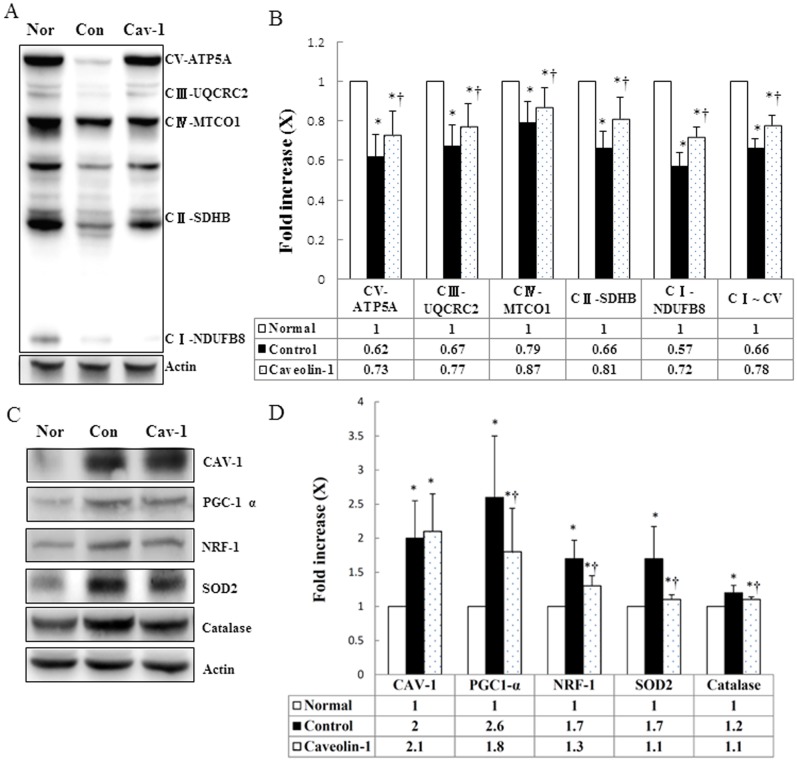
CAV-1 restored mitochondrial function, anti-oxidative capacity and mitochondrial respiratory chain subunits. (A) and (B), Immunoblots and densitometry of the mitochondrial complex subunits (*I*, *II*, *III*, *IV* and *V*) from 8-wk normal or high-cholesterol fed rabbits with or without CAV-1 treatment. (C) Representative immunoblot showing levels of mitochondrial biogenesis marker and antioxidant enzymes in normal rabbits, untreated rabbits, and treated hypercholesterolaemic rabbits. (D) Columns represent average values over three independent experiments. Density from the normal group was set as 1. * and † are significantly different from the normal and control groups, respectively, *P*<.05. β-actin was used as a loading control. Values are means ± SD.

To complement our quantification analysis, we used immunoblot analysis to measure mitochondrial OXPHOS complexes and anti-oxidative capacity. We found that the expression levels of ATP synthase α-subunit (complex V, +17.7%), NDUFB8 (complex I, +26.3%), SDHB (complex II, +22.7%), core 2 protein (complex III, +14.9%), and MTCO1 (complex IV, +10.1%) were significantly higher in the AP-CAV-1-treated group than in the untreated group (*P*<0. 05). In addition, AP-CAV-1 treatment resulted in significantly higher levels of expression of electron transport chain (ETC) complex I∼V proteins (+18.2%, *P*<0. 05), which suggests that CAV-1 treatment enhances mitochondrial function in animals with hypercholesterolemia (Figure 5A and 5B). We also found that the expression levels of proteins related to mitochondrial biogenesis (PGC-1 α, −30.8%; NRF-1, −23.5%) and anti-oxidation (SOD2, −35.3%; CAT, −8.3%) were significantly lower in the AP-CAV-1-treated group than in the untreated control group (*P*<0.05). CAV-1 protein expression also was higher in the CAV-1 group (+5.0%) (Figure 5C and 5D). These results are consistent with those obtained in the immunohistochemistry analysis.

### Apoptotic Cell Death

**Figure 6:**
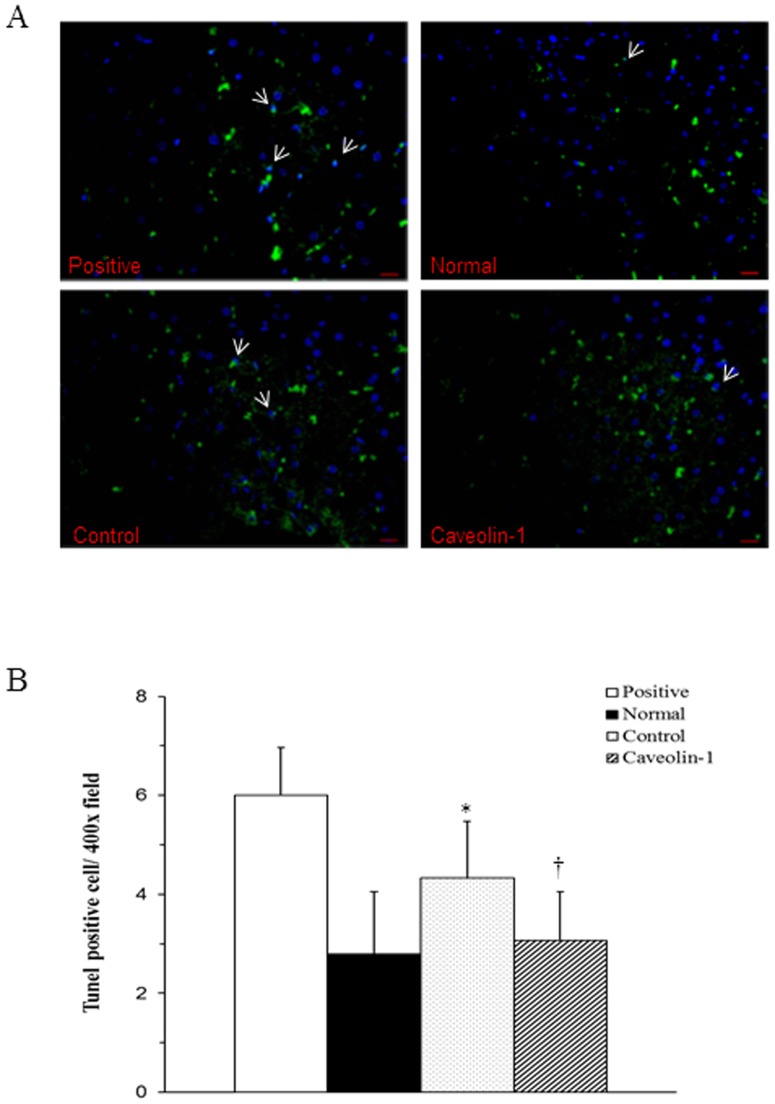
Terminal deoxynucleotidyl transferase-mediated dUTP nick-end labelling (TUNEL) assay and Hoechst staining analysed by immunofluorescence. (A) Signals of TUNEL-positive cells (arrows), which were upregulated when the rabbits were fed a high-cholesterol diet for 8 weeks, were higher in the control group than in the normal and CAV-1 groups. TUNEL staining has green fluorescence (Alexa Fluror 488nm), and the nuclei are stained with Hoechst dye (blue). (B) Quantification of TUNEL IHF stain in rabbit liver tissue per ×400 field/7 fields per animal, n = 6 rabbits for each group. A significantly suppressed number of TUNEL-positive cells were observed in the CAV-1 group. * and † are significantly different from the normal and control groups, respectively, *P*<.01. Values are mean ± SD from at least 3 independent experiments, n = 6 for each group.

We measured apoptotic cell death in hepatic cells using the TUNEL assay to investigate whether CAV-1 activity is related to cell survival. We found that the number of TUNEL-positive cells per high power field (400×) was significantly higher in the control group (4.33±1.14) than in the normal-diet group (2.79±1.26) or in the AP-CAV-1-treated group (3.07±0.98) (*P*<0.01) as shown in Figure 6A, indicating that treatment with CAV-1 significantly suppressed apoptosis. Hoechst staining showed the appearance of the nucleus. Quantification of the results is shown in Figure 6B.

## Discussion

In our novel study, we found that CAV-1-treated hypercholesterolemic rabbits had markedly less fat acumination in the liver than untreated animals. In addition, we also found that CAV-1 affects the molecular mechanisms governing mitochondrial biogenesis and the OXPHOS chain, as well as oxidative and anti-oxidative homeostasis, fatty acid oxidation, and apoptosis of hepatic cells.

Hypercholesterolemia is an important inducer of atherosclerosis and exclusion of excess cholesterol from cells and tissues can prevent the development of the disease. Cholesterol efflux involves a number of transporters including CAV-1, ATP-binding cassette (ABC) A1, ABCG1, SR-B1, and CTP 27A1 [38]. In 2001, Frank *et al*. demonstrated that CAV-1 downregulation enhanced cellular cholesterol efflux to HDL [10]. Similarly, our previous study provided evidence that CAV-1 levels in plaques located in coronary arteries and aortic arches of rabbits fed a high-cholesterol diet decreased after 8 weeks [37]. We also found that CAV-1 overexpression in aortic endothelial cells upregulated ABCA1 expression and enhanced HDL-mediated cholesterol efflux [38]. In this study, we revealed that AP-CAV-1 treatment helps restore LDL-C to normal levels and may provide palliation for adverse hepatic reactions by decreasing AST and ALT levels in hypercholesterolemic rabbits.

Although the effects of CAV-1 on intracellular cholesterol metabolism and cholesterol efflux have been studied previously, the results were inconsistent. Earlier studies revealed that significant increases in CAV-1-mediated transport of newly synthesized cholesterol to plasma membrane caveolae resulted in enhanced cholesterol efflux in L1210-JF cells [40], human fibroblasts [41], and THP-1 cells [42]. In contrast, other studies showed that changes in CAV-1 levels did not result in changes in cholesterol efflux in J774, RAW [43], HEK-293T, FRT [6], [44], and RAW 264.7 cells [12]. The reason for the observed differences is that the role of CAV-1 in cholesterol metabolism and efflux are cell-type specific. Studies have shown that the liver is a major source of HDL-C in plasma and that it may regulate reverse cholesterol transport by being both the origin and destination of the pathway [45]. CAVs are cholesterol-binding proteins that participate in many important cellular processes, including cholesterol transport. However, only a few studies have explored the role of CAVs in the liver. Moreno *et al.*, for example, found that hepatic CAV overexpression increases bile flow, bile salt-dependent biliary lipid secretion, plasma HDL-C levels, and hepatic free cholesterol levels in mice [11]. Fu *et al.*, also demonstrated that CAV-1 expression enhances cholesterol efflux in hepatic cells [12]. However, the molecular mechanisms governing these effects remain unclear.

Mitochondrial dysfunction plays a central role in the development of cholestatic liver diseases because mitochondria regulate cell death signalling - triggered by inflammatory cytokines or bile acid secretion - and contribute to oxidative damage and metabolic disorder [31]. Bosch *et al.*, have shown that CAV-1 participates in the regulation of mitochondrial cholesterol levels and that there is a connection between CAV, cholesterol metabolism, and mitochondrial diseases [13], [14]. In this study, we provide evidence that CAV-1 overexpression protects against fatty acid oxidation, increases mitochondrial OXPHOS chain function, suppresses the expression of Romo1 without increasing SOD2 and catalase activities, attenuates PGC-1α and NRF-1 expression, and protects against apoptosis.

There are some limitations in our study. First, the hypercholesterolemic rabbit model used in this study might not accurately reflect the relevant pathways in humans. Further studies should be conducted using other model species. Second, we did not perform time-dependent studies to evaluate whether mitochondria are the cause of the observed effects.

In summary, our results indirectly indicate that CAV-1 mediates the effects of PGC-1α on hepatic mitochondrial OXPHOS chain function, the antioxidant defence system, and mitochondrial biogenesis. Thus, CAV-1 may provide palliation for adverse hepatic reactions in hypercholesterolemic rabbits by attenuating the PGC-1α/NRF-1 pathway.
